# Rapid adjustments to autonomic control of cardiac rhythm at the onset of isometric exercise in healthy young adults

**DOI:** 10.14814/phy2.15616

**Published:** 2023-02-23

**Authors:** Tyler E. Oliver, Miguel E. Sánchez‐Hechavarría, Ramón Carrazana‐Escalona, Cheryl A. Blaha, Lawrence I. Sinoway, Rachel C. Drew

**Affiliations:** ^1^ Department of Exercise and Health Sciences University of Massachusetts Boston Boston Massachusetts USA; ^2^ Departamento de Ciencias Básicas, Facultad de Medicina Universidad Católica de la Santísima Concepción Concepción Chile; ^3^ Facultad de Ciencias de la Salud Universidad Adventista de Chile Chillán Chile; ^4^ Penn State Heart and Vascular Institute, Penn State College of Medicine Hershey Pennsylvania USA

**Keywords:** autonomic control, cardiac rhythm, exercise onset, heart rate variability, isometric exercise

## Abstract

Sympathetic nervous system (SNS) and parasympathetic nervous system (PNS) influences on cardiac rhythm at the onset of exercise, a time of rapid autonomic adjustments, are clinically important areas of investigation. Continuous wavelet transform (CWT) involves time‐frequency‐based heart rate variability (HRV) analysis allowing investigation of autonomic influences on cardiac rhythm during short durations of exercise. Therefore, the purpose of this study was to characterize SNS and PNS influences on cardiac rhythm at the onset of isometric exercise in healthy young adults. CWT analysis was retrospectively applied to R‐R interval data (electrocardiogram) previously collected from 14 healthy young adults (26 ± 2 years) who performed 30‐s, one‐legged, isometric, calf exercise at 70% maximal voluntary contraction (MVC; 70% MVC trial) or rested (0% MVC trial). Absolute and normalized low‐frequency (aLF, nLF; 0.04–0.15 Hz) and high‐frequency (aHF, nHF; 0.15–0.4 Hz) bands and LF/HF were used to analyze one 30‐s baseline period and six 5‐s time windows during the 30‐s exercise (70% MVC) or rest (0% MVC). Statistical analysis involved two‐way analysis of variance with post‐hoc analysis. aHF, aLF, LF/HF, nHF, and nLF displayed a trial‐time interaction (all *p* ≤ 0.027). In the 70% compared to the 0% MVC trial, aHF and nHF were lower after 5–30 s (all *p* ≤ 0.040), aLF was lower after 20–30 s (all *p* ≤ 0.011) and LF/HF and nLF were higher after 5–20 s (all *p* ≤ 0.045). These results indicate the reduction of the PNS influence on cardiac rhythm begins sooner than the augmentation of the SNS influence at the onset of isometric exercise in healthy young adults.

## Graphical abstract

Sympathetic and parasympathetic nervous system influences on cardiac rhythm at the onset of exercise, when rapid autonomic adjustments occur, are clinically important but under‐studied areas of investigation. Therefore, the focus of this paper was to assess sympathetic and parasympathetic nervous system influences on cardiac rhythm at the onset of isometric exercise in healthy young adults. The findings show that the reduction of the parasympathetic nervous system influence on cardiac rhythm begins sooner than the augmentation of the sympathetic nervous system influence at the onset of isometric exercise in healthy young adults, which provide important insight into the autonomic control of cardiac rhythm during acute physiological stress.

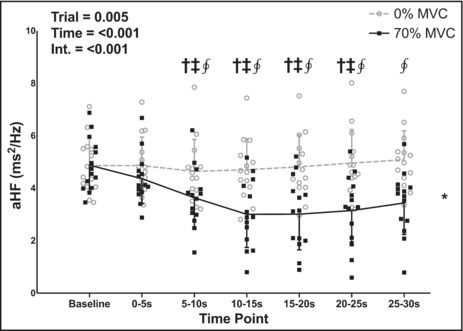

## 1 INTRODUCTION

The autonomic nervous system (ANS) is a mediator of cardiovascular adjustments to stimuli intrinsic and extrinsic to the body, and its relationship to cardiac health is well established (Bleich et al., 1976; Coumel, 1993; Shen & Zipes, 2014; Zhang & Anderson, 2014). The sympathetic nervous system (SNS) and parasympathetic nervous system (PNS) orchestrate many of the regulatory responses to fluctuations in physiological variables, such as heart rate (HR). It is often when the function of these autonomic branches is altered from aging or due to acute or chronic stress that diseases such as hypertension, congestive heart failure, or arrhythmias begin to develop or worsen (Shaffer & Ginsberg, 2017; Task Force Report, 1996).

One of the physiological parameters that indicates both cardiac health and responses to physiological stressors, such as exercise, is HR. Even among healthy individuals, there are complex displays of HR variability (HRV) that can indicate significant autonomic adjustments both at rest and during exercise that are needed to maintain homeostatic balance. In recording complex biological signals, there has been a large recognition of using HRV to quantify these autonomic adjustments and assess its overall impact on cardiac health and mortality (Akselrod et al., 1981; Bigger, 1997; Malliani et al., 1991; Saykrs, 1973; Thayer et al., 2010). During exercise, HR increases due to progressive PNS withdrawal and SNS activation as exercise intensity increases (Rowell & O'Leary, 1990; White & Raven, 2014). Therefore, the time between successive beats shortens and overall HRV decreases during exercise. In quantifying HRV, the time intervals between successive beats over short‐term (5 min) or long‐term (24 h) electrocardiogram (ECG) recordings can be measured, hence time‐domain analysis and a sense of overall variability in HR for a period in a stationary ECG signal. Like time‐domain analysis, oscillations in the HRV signal can be captured by frequency‐domain analysis in which component wavelengths (high frequency (HF) and low frequency (LF)) can be measured and provide insights into cardiac autonomic activity. It has been widely accepted that PNS activity is reflected in higher frequencies, whereas identification of SNS activity in lower frequencies and the ratio of LF/HF is more controversial (Houle & Billman, 1999; Shaffer & Ginsberg, 2017; Task Force Report, 1996). While both techniques provide useful information about HRV, neither can identify instantaneous changes in HRV from rapid autonomic adjustments during acute physiological stressors. Continuous wavelet transform (CWT) is a form of HRV analysis based on time‐frequency analysis that can localize shifts in HRV instantaneously across time. This instantaneous and continuous extraction of temporal and spectral information can be a much more sensitive analysis tool for understanding autonomic regulation of cardiac rhythm, especially during a time of rapid autonomic adjustments.

Investigations into autonomic regulation of cardiac rhythm during exercise of various intensities have been conducted (Cottin et al., 2004; Fontolliet et al., 2018; Martinmäki et al., 2008; Tulppo et al., 1996; Yamamoto et al., 1991). During light‐intensity exercise, normalized LF power increases and normalized HF power decreases compared to resting levels, resulting in greater LF/HF during exercise compared to rest, indicating a shift from predominantly parasympathetic control of cardiac rhythm at rest to increased sympathetic control of cardiac rhythm during light‐intensity exercise (Cottin et al., 2004; Fontolliet et al., 2018). Greater normalized LF power was observed during moderate‐intensity exercise, yet a greater normalized HF power was observed during high‐intensity exercise above ventilatory threshold (Cottin et al., 2004). Findings from these investigations are somewhat discrepant, highlighting the complexity of assessing HRV during exercise when data are non‐stationary or undergoing rapid fluctuations. The onset of exercise is a time of rapid autonomic adjustments, and the contributions of SNS and PNS activities to cardiac rhythm at this time have not been investigated. Investigating the autonomic influences on HRV during short time windows may be clinically relevant to populations with cardiovascular disease or autonomic dysfunction, as short bouts of physical activity occur more frequently during activities of daily living than longer, dedicated periods of exercise. Hence, the purpose of this study was to characterize SNS and PNS influences on cardiac rhythm at the onset of isometric exercise in healthy young adults to identify the autonomic adjustments occurring during this time of rapid changes in autonomic control. It was hypothesized that the overall SNS influence on cardiac rhythm, measured by LF and LF/HF, would increase, while the overall PNS influence on cardiac rhythm, measured by HF, would decrease, at the onset of exercise in healthy young adults.

## 2 METHODS

### 2.1 Ethics approval

This study was based on a retrospective analysis of previously collected data. The experimental protocol in which the data were previously collected was approved by the Institutional Review Board of the Penn State Milton S. Hershey Medical Center and conformed to the Declaration of Helsinki (#35884). Subjects provided written informed consent after the purpose of, and risks involved in, the protocol were explained to them. This protocol took place in the Clinical Research Center of Penn State Milton S. Hershey Medical Center.

### 2.2 Subjects

Previously collected data from 14 healthy young adults (Table 1) were retrospectively analyzed using CWT HRV analysis. All subjects were normotensive, recreationally active, not taking any medications, and did not have any known history of cardiovascular or autonomic conditions. Subjects were asked to avoid eating for 8 h, as well as ingesting caffeine or alcohol and performing exercise for 24 hours prior to performing the protocol.

**TABLE 1:** Demographic characteristics, baseline cardiovascular values, and maximal voluntary contraction values of subjects.

Variable	Mean ± SD
Age (years)	26 ± 2
Sex	6 male/8 female
Race/ethnicity	12 White, NH/ 2 AA, NH
Height (cm)	174 ± 11
Weight (kg)	75 ± 16
Body mass index (kg.m^−2^)	24.4 ± 2.7
Systolic blood pressure (mmHg)	113 ± 9
Diastolic blood pressure (mmHg)	72 ± 6
Mean arterial blood pressure (mmHg)	86 ± 7
Heart rate (b.min^−1^)	62 ± 8
Maximum voluntary contraction (Nm)	102 ± 39

Abbreviations: AA, African American; NH, non‐Hispanic; SD, standard deviation.

Baseline data for systolic blood pressure (BP), diastolic BP, mean arterial BP, HR, and maximal voluntary contraction (MVC) from 10 of these 14 young subjects were included in analyses in previously published studies (Drew et al., 2013, 2017). The focus of the current study was the SNS and PNS contributions to the control of cardiac rhythm at the onset of isometric exercise in healthy young adults, which has not been investigated to date. Novel data on HRV parameters (aLF, aHF, nLF, nHF, and LF/HF) and R‐R intervals (RRI) during baseline and all HRV parameters, HR, and RRI during the first 30 s of isometric exercise performance from all 14 young subjects are presented here. Baseline data for systolic BP, diastolic BP, mean arterial BP, HR, and MVC are also included to illustrate the baseline cardiovascular and exercise values related to the exercise responses that were observed.

### 2.3 Experimental protocol

**FIGURE 1:**
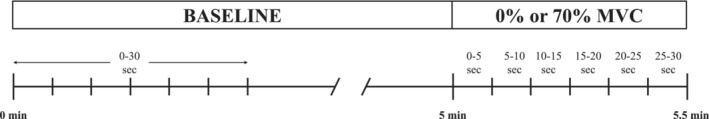
A schematic diagram of the experimental protocol showing a 5‐min resting baseline for each trial followed by either 70% maximal voluntary contraction (MVC), isometric, calf exercise performance, or continued rest (0% MVC) for 30 s. Data were analyzed in 5‐s time intervals during baseline and the rest/exercise phase of each trial, with the baseline data then averaged to produce one 30‐s average for the baseline period. Min, minute; MVC, maximal voluntary contraction. Timeline is not to scale.

Subjects attended one experimental visit, performing two trials within this visit. Subjects were seated in a semi‐supine position with their right leg flexed by 30° with the lower leg parallel to the ground and the foot strapped to a footplate to minimize heel lift during calf exercise. MVC of the right calf plantarflexor muscles was measured by recording the maximal torque that was produced when subjects briefly pushed as hard as possible against the footplate. After repeating this effort three‐to‐five times, each effort separated by ~1 min, the largest torque recording was used as the subject's MVC. Seventy percent of each subject's MVC was calculated and set on a torque display box positioned in front of the subject, so they could visualize the level of torque produced during exercise in the 70% MVC trial. After subjects were settled for at least 10 min after completion of the experimental set‐up, the trial began with a 5‐min baseline phase. Following this resting phase, subjects then either performed isometric, right calf exercise at 70% MVC for 30 s (70% MVC trial) or continued to rest (0% MVC control trial) (Figure 1). Performance of these trials was separated by ~20 min to ensure that baseline hemodynamics were restored after performance of the first trial and before performance of the second trial. The order of these two trials was counterbalanced within each visit across all subjects.

### 2.4 Experimental measurements

Three baseline BP measurements were taken before each trial using a semi‐automated arm cuff (SureSigns VS3; Philips) to provide resting cardiovascular values of participants. RRIs were continuously measured using a three‐lead ECG (Cardiocap/5; GE Healthcare), from which HR was derived, to allow assessment of heart rate variability. Respiratory movements were continuously measured using a pneumography belt placed around the abdomen to confirm that participants were breathing appropriately during the trials. Torque levels produced during calf exercise were measured using a load cell on the footplate. An analog‐to‐digital converter (Cambridge Electronic Design (CED) 1401plus; CED, Cambridge, UK) was used to sample the ECG, pneumograph, and torque signals, with signals sampled at 1000 Hz. Data were recorded and displayed during the trials and analyzed offline using Spike2 software (CED; RRID:SCR_000903).

### 2.5 Data and statistical analyses

CWT was applied to the RRI data for HRV analysis using Matlab 2016b software (MathWorks Inc.; RRID:SCR_001622). CWT has been proposed as an advantageous method for analyzing HRV in situations in which rapid changes in ANS activity occur because this approach allows for temporal evolution across the HRV recording and precise localization of spectral power to identify acute shifts in ANS activity (Belova et al., 2007; Pichot et al., 1999). Due to the wavelet shape used in this data analysis, termed scale factors, CWT can be applied in ways that better fit the shape of the signal, allowing for better quantitative measurement compared to traditional frequency‐domain HRV analysis. CWT involves using every possible wavelet within the LF (0.04–0.15 Hz) and HF (0.15–0.40 Hz) bands to extract frequency information instantaneously at any time point (Belova et al., 2007; Cartas‐Rosado et al., 2020; Wiklund et al., 1997). Raw data files were analyzed using custom‐written script files to produce RRI values using Spike2 software (CED; RRID:SCR_000903). Prior to CWT analysis, pre‐processing of RRI data involved removal of ectopic beats and detrending. To obtain HRV measures using instantaneous power methods, the squared modulus of the wavelet coefficients was integrated over the desired frequency band [f1 f2]. These wavelet coefficients correspond to the specific scale factor applied to the RRI data from the ECG signal. To integrate power, frequency band wavelet scales were changed to frequencies. The values of power of each frequency band of the HRV in time were interpolated to 4 Hz, meaning frequency information was extracted from the signal instantaneously every 0.25 s (four data points per second). The instantaneous power of the frequency band [f1 f2] is given by:

$$P_{\mathrm{CWT}}\left(t\right)=\frac{1}{C_{\psi }}\int_{\alpha_{1}}^{\alpha_{2}}{\left|W\left(t,\alpha \right)\right|}^{2}\frac{d\alpha }{\alpha^{2}}=\frac{1}{C_{\psi }f_{\psi }}\int_{f_{1}}^{f_{2}}{\left|W\left(t,\frac{f_{\psi }}{f}\right)\right|}^{2}df$$

RRI data were analyzed using LF (0.04–0.15 Hz) and HF (0.15–0.4 Hz) bands for HRV analysis. These LF and HF components are reported as log‐transformed absolute power expressed in ms^2^/Hz (aLF, aHF), and normalized values (nLF, nHF) where nLF = (aLF)/(aLF + aHF) and aHF = (aHF)/(aLF + aHF). In addition, the ratio of the aLF and aHF values was calculated (LF/HF). The first six 5‐s time windows at the start of the 5‐min baseline period were analyzed and then averaged to provide a 30‐s baseline value for analysis. This initial baseline period (0–30 s) was chosen, as opposed to the final 30 s of the 5‐min baseline period (270–300 s), to avoid the influence of anticipation of upcoming exercise performance in the 70% MVC trial causing HR to increase prior to starting exercise performance (McArdle et al., 1967). The six 5‐s time windows during the 30‐s rest/exercise phase of each trial were analyzed. As frequency information is instantaneously extracted from the signal using the CWT approach, these 5‐s time windows provide sufficient time resolution despite the brevity of their duration, highlighting the advantageous nature of this time‐frequency‐based CWT analysis approach for assessing HRV (Davrath et al., 2006).

The normality of the data for all five variables (aLF, aHF, nLF, nHF, and LF/HF) was tested using the Shapiro–Wilk test. For each variable, data were plotted on a box‐and‐whisker plot, and any outliers were identified as values falling outside 1.5 times the interquartile range above the upper quartile or below the lower quartile. Following normality testing, data from all 14 subjects were included in data analysis. Group mean and standard deviation values for data for all five variables were calculated for each time period (one baseline time period – 0‐30 s, and six rest/exercise time periods – 0‐5, 5–10, 10–15, 15–20, 20–25 and 25–30 s) in each trial (0% and 70% MVC). Baseline data from the 0% and 70% MVC trials for all variables were compared using a paired *t* test. A two‐way, repeated measures, analysis of variance was conducted on data for all variables, with within‐subject factors of trial (two levels) and time (seven levels across two phases). Post‐hoc analysis was conducted when main effects for trial, time, and/or an interaction between these factors were identified, which involved paired *t* tests and a Holm–Bonferroni correction. Statistical significance was set at *p* < 0.05 for all analyses, and all statistical analyses were performed using SPSS Statistics for Windows (IBM; RRID:SCR_016479), with figures created using Prism (GraphPad; RRID:SCR_002798). Additionally, while the purpose of this manuscript was to characterize SNS and PNS influences on cardiac rhythm at the onset of isometric exercise in healthy young adults without specifically investigating potential sex differences, graphical data are also presented disaggregated by sex to display any potential sex differences in the responses observed.

## 3 RESULTS

### 3.1 Heart rate and R‐R interval

**FIGURE 2:**
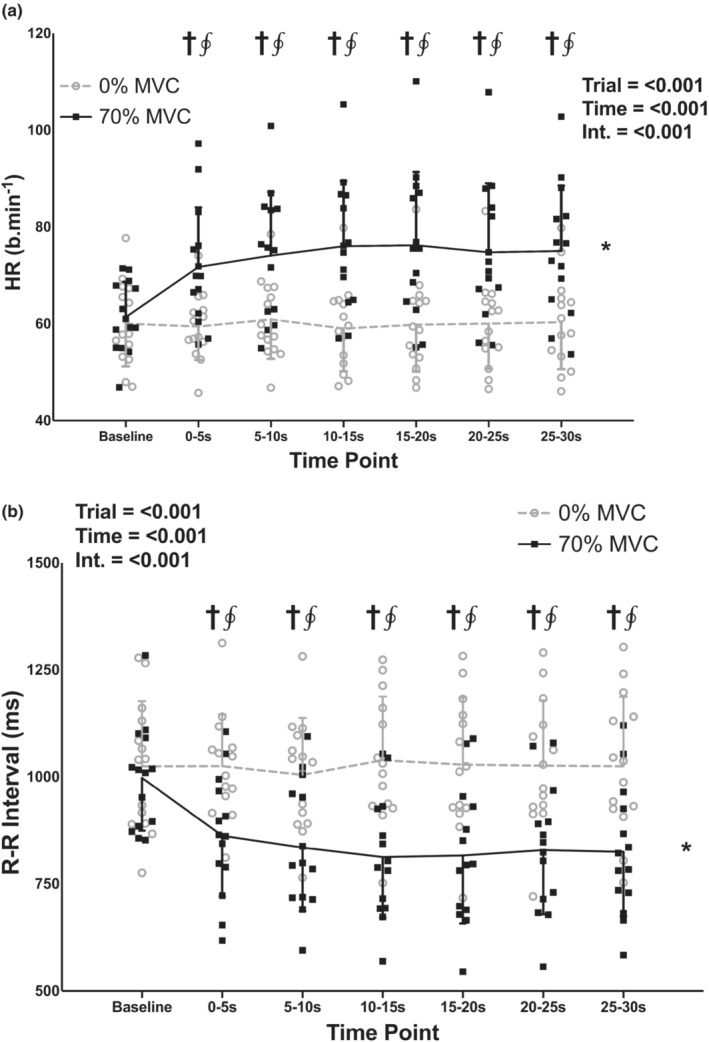
Individual subject data and group means ± standard deviations for heart rate (HR; a) and R‐R intervals (RRI; b) during baseline and 30‐s, one‐legged, isometric, calf exercise at 70% maximal voluntary contraction (MVC; 70% MVC trial) or continued rest (0% MVC trial). Statistical analysis involved two‐way, repeated measures, analysis of variance with post‐hoc analysis involving paired *t* tests and a Holm–Bonferroni correction as appropriate. Int, interaction. *Significantly different from 0% trial. ^†^Significantly different from baseline (all *p* < 0.05). ^∮^Significantly different from 0% at specific time point (all *p* < 0.05).

There were no differences in baseline values between the 0% and 70% MVC trials for HR and RRI (*p* = 0.326 and 0.289, respectively). There was a significant effect of trial for both HR and RRI (Figure 2; both *p* < 0.001), with HR being higher and RRI being lower in the 70% compared to the 0% MVC trial. There was also a significant effect of time for both HR and RRI (both *p* < 0.001), with the 0–5, 5–10, 10–15, 15–20, 20–25, and 25–30‐s time points being higher than the baseline time point for HR (*p* = 0.021, 0.009, 0.015, 0.031, 0.032, and 0.010, respectively) and lower than the baseline time point for RRI (*p* = 0.003, 0.002, 0.010, 0.013, 0.007, and 0.004, respectively). There was also a significant trial‐time interaction for both HR and RRI (both *p* < 0.001), with HR being higher and RRI being lower in the 70% compared to the 0% MVC trial at the 0–5, 5–10, 10–15, 15–20, 20–25, and 25–30‐s time points (all *p* = 0.006).

### 3.2 Absolute HRV power

**FIGURE 3:**
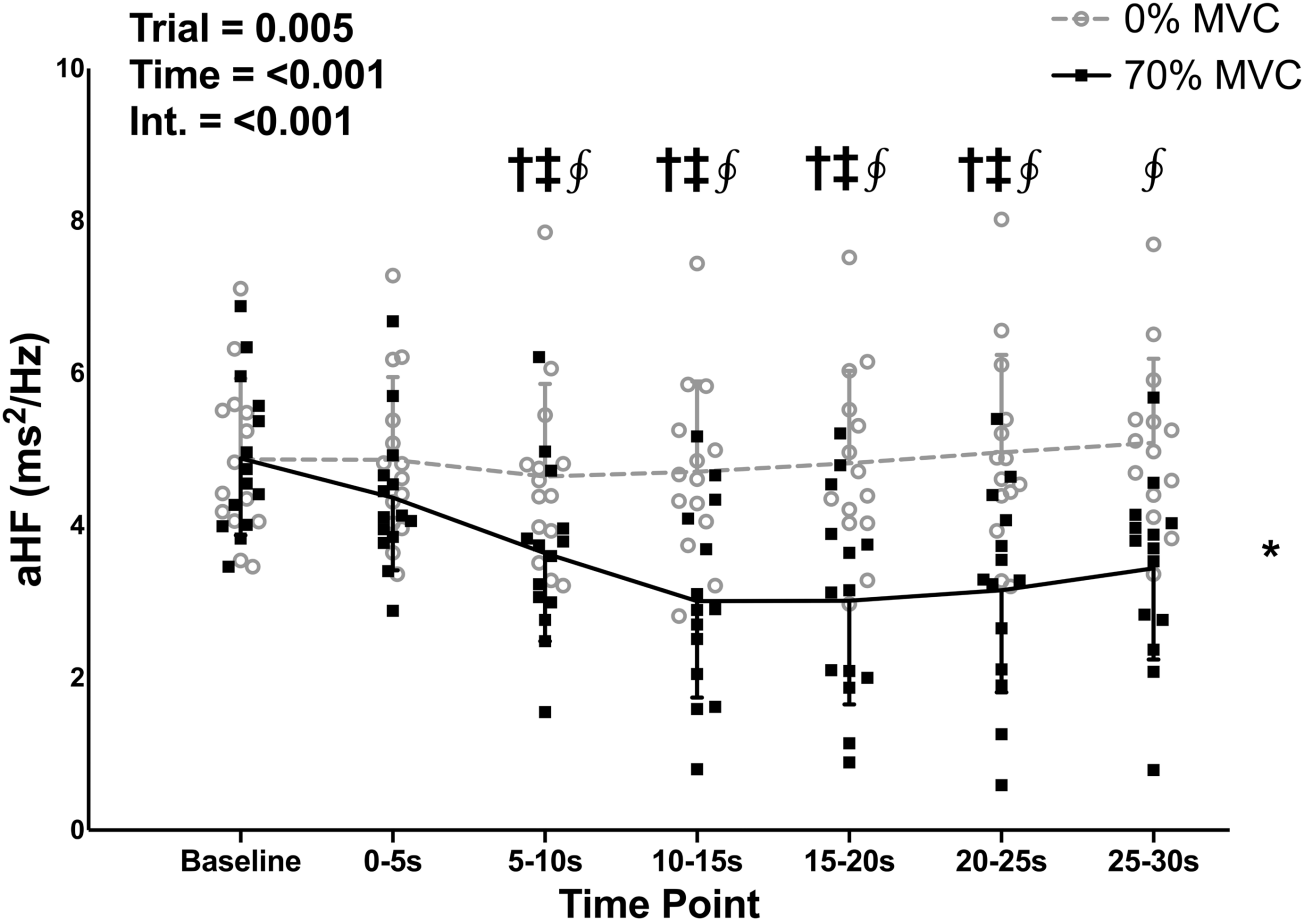
Individual subject data and group means ± standard deviations for absolute high frequency (aHF) power during baseline and 30‐s, one‐legged, isometric, calf exercise at 70% maximal voluntary contraction (MVC; 70% MVC trial) or continued rest (0% MVC trial). Statistical analysis involved two‐way, repeated measures, analysis of variance with post‐hoc analysis involving paired *t* tests and a Holm–Bonferroni correction as appropriate. Int, interaction. *Significantly different from 0% trial. ^†^Significantly different from baseline (all *p* < 0.05). ^‡^Significantly different from 0–5 s (all *p* < 0.05). ^∮^Significantly different from 0% at specific time point (all *p* < 0.05).

**FIGURE 4:**
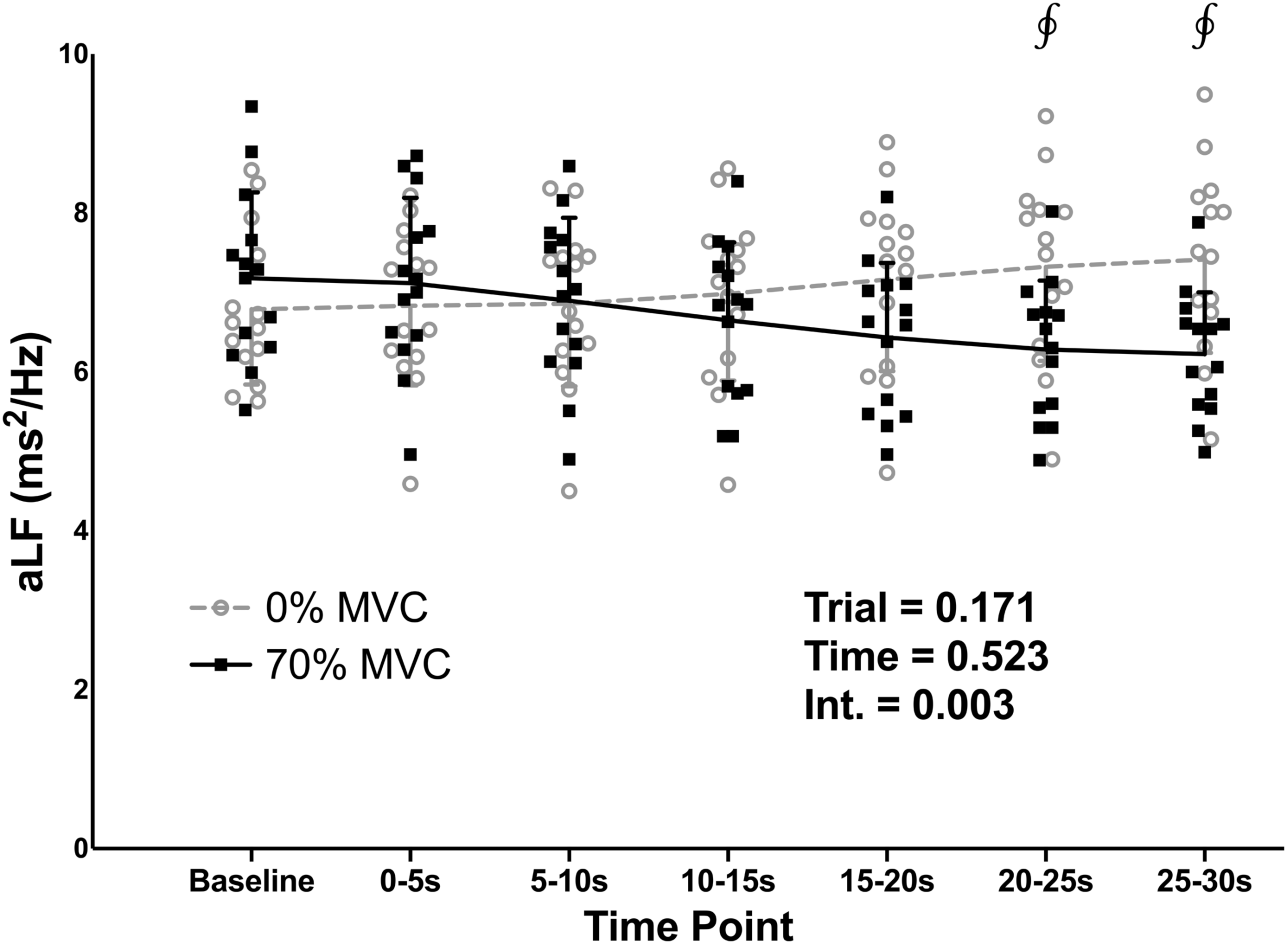
Individual subject data and group means ± standard deviations for absolute low frequency (aLF) power during baseline and 30‐s, one‐legged, isometric, calf exercise at 70% maximal voluntary contraction (MVC; 70% MVC trial) or continued rest (0% MVC trial). Statistical analysis involved two‐way, repeated measures, analysis of variance with post‐hoc analysis involving paired *t* tests and a Holm–Bonferroni correction as appropriate. Int, interaction. ^∮^Significantly different from 0% at specific time point (*p* < 0.05).

There were no differences in baseline values between the 0% and 70% MVC trials for aHF, aLF, and LF/HF (*p* = 0.934, 0.188, 0.458, respectively). There was a significant effect of trial for aHF (Figure 3; *p* = 0.005), with aHF being lower in the 70% compared to the 0% MVC trial. There was also a significant effect of time for aHF (*p* < 0.001), with the 5–10, 10–15, 15–20, and 20–25‐s time points being lower than the baseline (*p* = 0.036, 0.009, 0.015, and 0.009, respectively) and 0–5‐s (*p* = 0.029, 0.005, 0.015, and 0.036, respectively) time points. There was a significant trial‐time interaction for both aHF and aLF (*p* < 0.001 and *p* = 0.003, respectively). aHF was lower in the 70% compared to the 0% MVC trial at the 5–10, 10–15, 15–20, 20–25, and 25–30‐s time points (*p* = 0.025, 0.012, 0.012, 0.012, and 0.010, respectively). aLF (Figure 4) was lower in the 70% compared to the 0% MVC trial at the 20–25 and 25–30‐s time points (*p* = 0.011 and 0.008, respectively).

**FIGURE 5:**
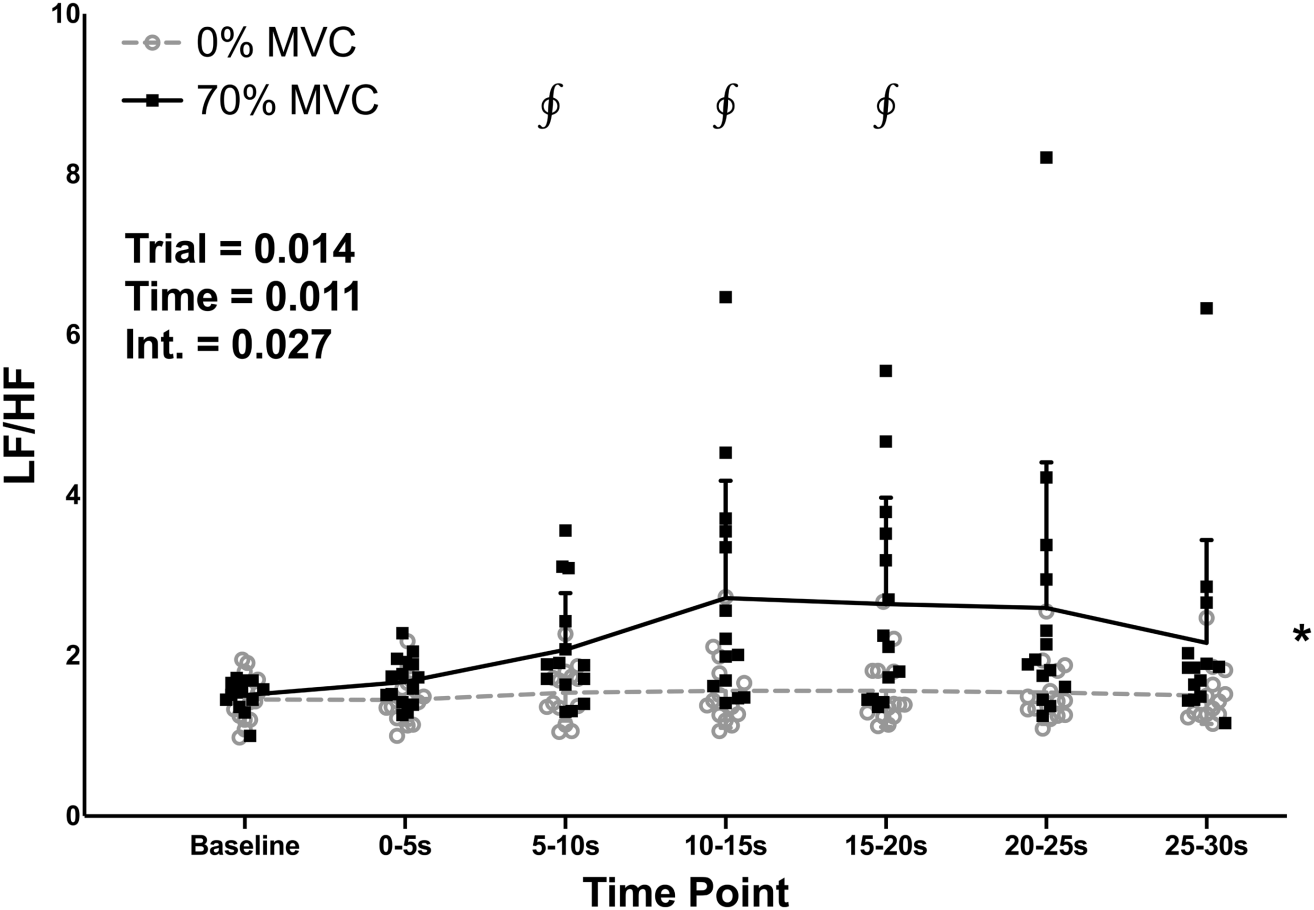
Individual subject data and group means ± standard deviations for low frequency (LF)/high frequency (HF) during baseline and 30‐s, one‐legged, isometric, calf exercise at 70% maximal voluntary contraction (MVC; 70% MVC trial) or continued rest (0% MVC trial). Statistical analysis involved two‐way, repeated measures, analysis of variance with post‐hoc analysis involving paired *t* tests and a Holm–Bonferroni correction as appropriate. Int, interaction. *Significantly different from 0% trial. ^∮^Significantly different from 0% at specific time point (all *p* < 0.05).

There was a significant effect of trial for LF/HF (Figure 5; *p* = 0.014), with LF/HF being higher in the 70% compared to the 0% MVC trial. There was also a significant effect of time (*p* = 0.011) and a significant trial‐time interaction (*p* = 0.027) for LF/HF. LF/HF was higher in the 70% compared to the 0% MVC trial at the 5–10, 10–15, and 15–20‐s time points (*p* = 0.045, 0.040, and 0.040, respectively).

### 3.3 Normalized HRV power

**FIGURE 6:**
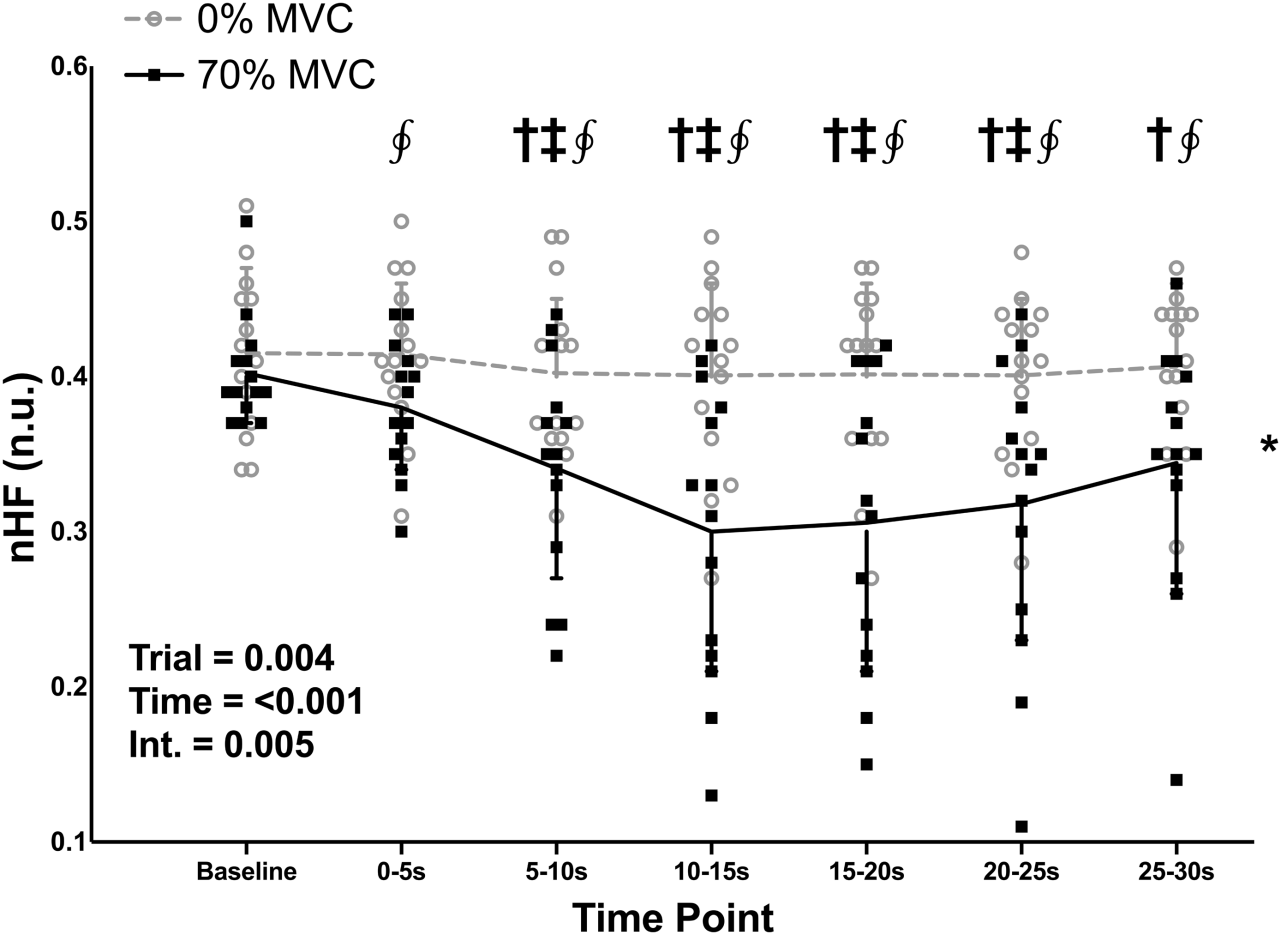
Individual subject data and group means ± standard deviations for normalized high frequency (nHF) power during baseline and 30‐s, one‐legged, isometric, calf exercise at 70% maximal voluntary contraction (MVC; 70% MVC trial) or continued rest (0% MVC trial). Statistical analysis involved two‐way, repeated measures, analysis of variance with post‐hoc analysis involving paired *t* tests and a Holm–Bonferroni correction as appropriate. n.u., normalized units. Int, interaction. *Significantly different from 0% trial. ^†^Significantly different from baseline (all *p* < 0.05). ^‡^Significantly different from 0–5 s (all *p* < 0.05). ^∮^Significantly different from 0% at specific time point (all *p* < 0.05).

**FIGURE 7:**
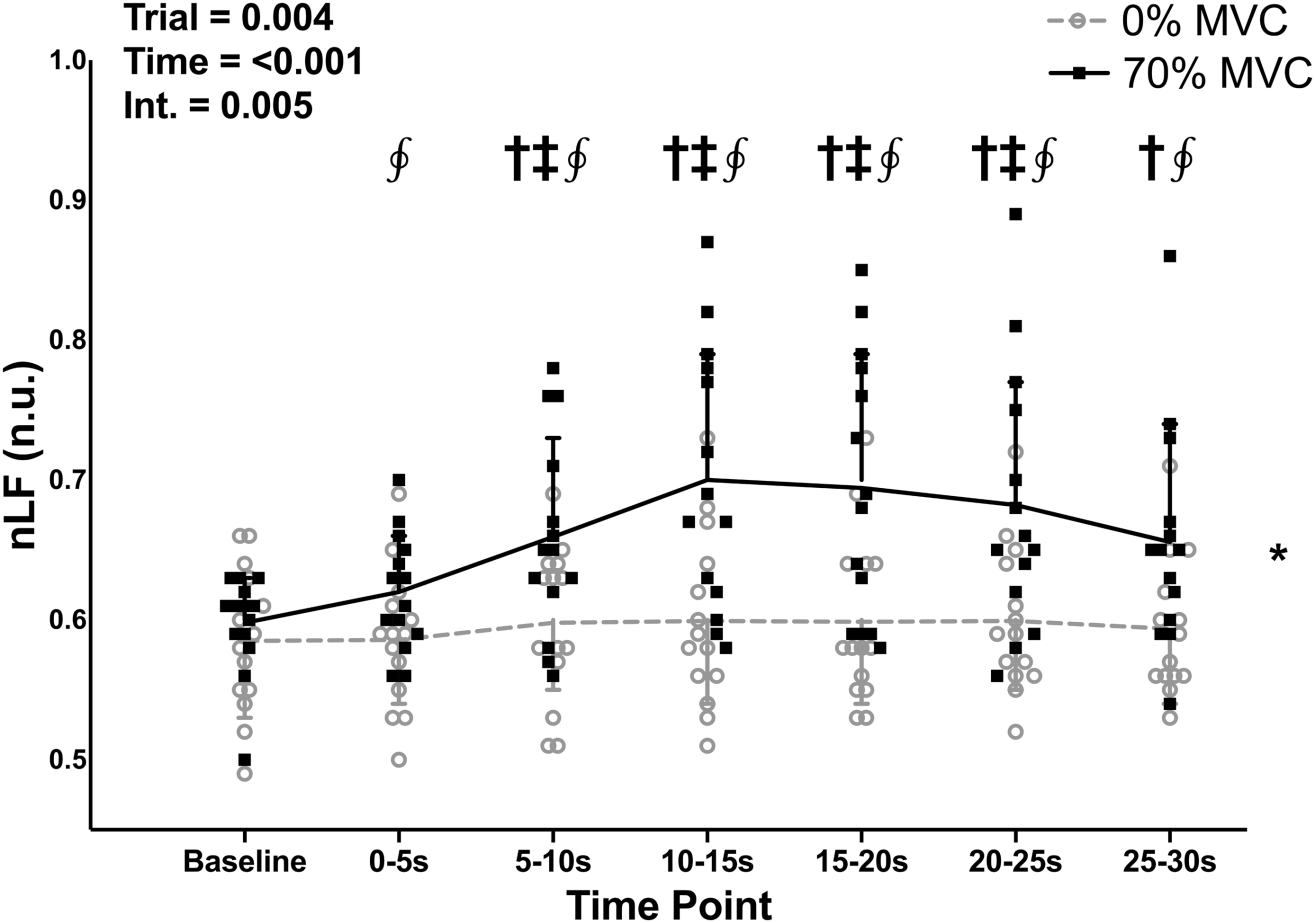
Individual subject data and group means ± standard deviations for normalized low frequency (nLF) power during baseline and 30‐s, one‐legged, isometric, calf exercise at 70% maximal voluntary contraction (MVC; 70% MVC trial) or continued rest (0% MVC trial). Statistical analysis involved two‐way, repeated measures, analysis of variance with post‐hoc analysis involving paired *t* tests and a Holm–Bonferroni correction as appropriate. n.u., normalized units. Int, interaction. *Significantly different from 0% trial. ^†^Significantly different from baseline (all *p* < 0.05). ^‡^Significantly different from 0–5 s (all *p* < 0.05). ^∮^Significantly different from 0% at specific time point (all *p* < 0.05).

There were no differences in baseline values between the 0% and 70% MVC trials for nHF and nLF (*p* = 0.291, both). There was a significant effect of trial for both nHF and nLF (Figures 6 and 7; both *p* = 0.004), with nLF being higher and nHF being lower in the 70% compared to the 0% MVC trial. There was also a significant effect of time for both nHF and nLF (both *p* < 0.001), with the 5–10, 10–15, 15–20, 20–25, and 25–30‐s time points being higher than the baseline time point for nLF (*p* = 0.009, 0.002, 0.002, 0.002, and 0.018, respectively) and lower than the baseline time point for nHF (*p* = 0.009, 0.002, 0.002, 0.002, and 0.018, respectively). There was also a significant trial‐time interaction for both nLF and nHF (both *p* = 0.005), with nLF being higher and nHF being lower in the 70% compared to the 0% MVC trial at the 0–5, 5–10, 10–15, 15–20, 20–25, and 25–30‐s time points (both *p* = 0.040, 0.040, 0.012, 0.020, 0.040, and 0.040 for each time point, respectively).

### 3.4 Potential sex differences in HRV power

**FIGURE 8:**
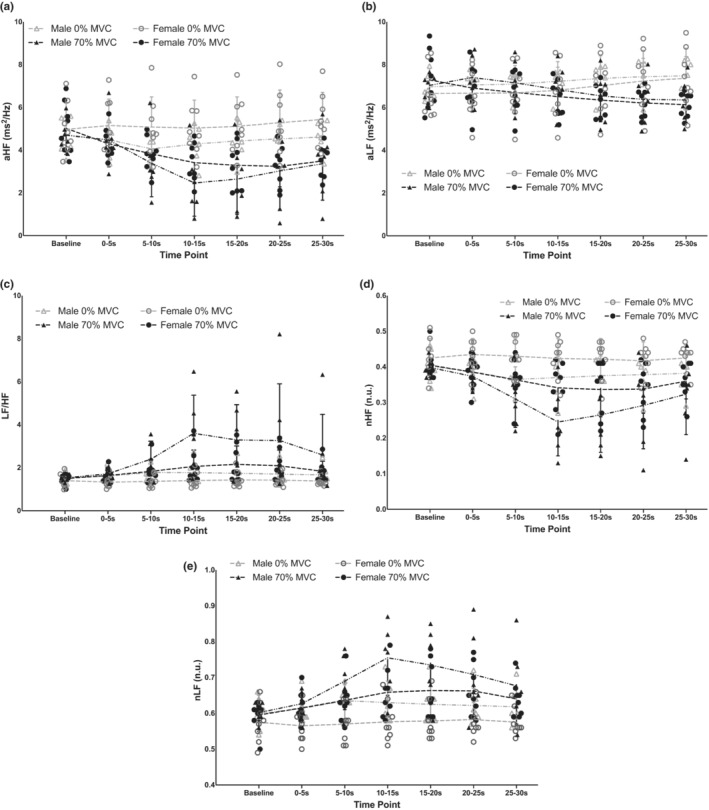
Individual subject data and group means ± standard deviations for absolute high frequency (aHF; a), absolute low frequency (aLF; b), low frequency/high frequency ratio (LF/HF; c), normalized high frequency (nHF; d) and normalized low frequency (nLF; e) during baseline and 30‐s, one‐legged, isometric, calf exercise at 70% maximal voluntary contraction (MVC; 70% MVC trial) or continued rest (0% MVC trial) disaggregated by sex. n.u., normalized units.

Data for aHF, aLF, LF/HF, nHF, and nLF disaggregated by sex are presented in Figure 8.

## 4 DISCUSSION

### 4.1 Summary of findings

The main findings of this study are that aHF, aLF, and nHF decrease from baseline levels and LF/HF and nLF increase from baseline levels within the first 30 s of the onset of isometric exercise in healthy young adults. Further, the decrease from baseline in aHF occurs earlier than the decrease from baseline in aLF at the onset of isometric exercise. Additionally, the increase in LF/HF occurs at the same time as the decrease in aHF, suggesting that aHF is the primary influence on LF/HF at this time compared to aLF. Overall, these results suggest that the reduction of the PNS influence on cardiac rhythm begins sooner than the augmentation of the SNS influence on cardiac rhythm at the onset of isometric exercise in healthy young adults.

### 4.2 Absolute HRV power at onset of isometric exercise

Findings from previous studies have shown similar decreases from resting levels in aLF and aHF power during exercise of increasing intensity (Cottin et al., 2004; Fontolliet et al., 2018; Martinmäki et al., 2008; Shiraishi et al., 2021; Tulppo et al., 1996; Yamamoto et al., 1991) and one‐legged, low‐intensity, isometric exercise (Weippert, Behrens, Gonschorek, et al., 2015). Further, LF/HF has been shown to either progressively decrease as exercise intensity increases (Cottin et al., 2004; Tulppo et al., 1996) or increase sharply during high‐intensity exercise in contrast to a lower level during lower‐intensity exercise (Shiraishi et al., 2021; Yamamoto et al., 1991). In the current study, both aHF and aLF decreased from baseline levels during the first 30 s of the onset of isometric exercise. aHF was significantly lower in the 70% compared to the 0% MVC trial from 5–10 s after the start of isometric exercise until the end of the 30‐s exercise period. aLF was lower in the 70% compared to the 0% MVC trial from 20–25 s after the start of isometric exercise until the end of the 30‐s exercise period. Therefore, the decrease in aHF from its baseline level occurred earlier than the decrease in aLF from its baseline level at the onset of exercise. As a result of the changes in aHF and aLF at the onset of isometric exercise, LF/HF was higher in the 70% compared to the 0% MVC trial from 5–20 s at the onset of isometric exercise.

From the instantaneous changes in the distribution of HRV parameters that were measured using CWT during the onset of isometric exercise in this study, aHF decreased steadily from baseline by ~38% at the 10–15‐s time point and plateaued at this lower level for the remainder of the 30‐s exercise period, while aLF decreased from baseline by only ~7% at the 10‐15‐s time point and continued to progressively decrease until the end of the exercise period. These responses of differing degrees and timings were reflected in LF/HF, as the ratio increased progressively from baseline by ~80% at the 10–15‐s time point and gradually decreased toward the baseline value during the remainder of the exercise period. Our findings indicate that the changes in aHF contributed more to the changes in LF/HF than the changes in aLF. Within the first 15 s of exercise, aHF displayed a relatively large effect on LF/HF, indicating a reduction in PNS activity and influence on HR regulation, supporting the hypothesis that the PNS influence on cardiac rhythm, measured by HF, would decrease at the onset of exercise in healthy young adults. These results are consistent with our current understanding of the autonomic response to exercise (Michael et al., 2017; White & Raven, 2014) regarding the reduced PNS activity through cardiac vagal withdrawal that occurs at the onset of exercise.

The decrease in aHF toward the start of the 30‐s exercise period was followed by a decrease in aLF toward the end of the 30‐s exercise period. These findings were expected and may be explained by the relationship between total power and HR, as during a period of sympathetic activation, that is, exercise, the consequent decrease in RRI and therefore increase in HR results in a reduction in total power for both absolute parameters (McCraty & Shaffer, 2015; Task Force Report, 1996). The overall increase in LF/HF at the onset of exercise supports the hypothesis that the SNS influence on cardiac rhythm would increase in healthy young adults. LF/HF was higher during the 70% compared to the 0% MVC trial and, specifically, LF/HF increased from its baseline level during the middle portion of the 30‐s exercise period. This higher level of LF/HF at the onset of isometric exercise was expected due to the decreased PNS and increased SNS activities resulting from central command and muscle mechanoreflex activation reflected in the HF and LF components, respectively (Cottin et al., 2004; Fontolliet et al., 2018; Rowell & O'Leary, 1990; White & Raven, 2014). However, the timing of the aLF decrease from its baseline level, which occurred after the decrease in aHF from its baseline level, suggests that the resultant increase in LF/HF was due more to the change in the PNS influence on cardiac rhythm rather than the SNS influence. The decrease in aLF toward the end of the 30‐s exercise period is in agreement with previous studies in which aLF decreases with increasing exercise intensity (Fontolliet et al., 2018; Martinmäki et al., 2008; Shiraishi et al., 2021; Tulppo et al., 1996; Yamamoto et al., 1991). The timing of the decreases in aHF and aLF and their resultant effects on LF/HF indicate that parasympathetic withdrawal is the primary influence on HRV at the onset of isometric exercise and that sympathetic activation may not substantially influence HRV during isometric exercise of as little as 30 s in duration. While findings from some previous studies suggest aLF includes both PNS and SNS influences (Akselrod et al., 1981; Shaffer & Ginsberg, 2017; Task Force Report, 1996), the current findings regarding aHF and therefore the PNS influence on cardiac rhythm at the onset of isometric exercise further illustrate the dynamic nature of autonomic influences on HRV during exercise.

### 4.3 Normalized HRV power at onset of isometric exercise

nHF was lower in the 70% compared to the 0% MVC trial, providing further support for the hypothesis that the PNS influence on cardiac rhythm, measured by HF, would decrease at the onset of isometric exercise in healthy young adults. This lower level of nHF at the onset of high‐intensity isometric exercise is in contrast to the greater nHF level previously reported during high‐intensity dynamic exercise (Cottin et al., 2004). This disparity could be explained by the different durations and/or modes of exercise involved in the two studies, with the current study using a 30‐s, isometric exercise period compared to a dynamic, incremental exercise test lasting at least several minutes. nLF was higher in the 70% compared to the 0% MVC trial, supporting the hypothesis that the SNS influence on cardiac rhythm, measured by LF, would increase at the onset of isometric exercise in healthy young adults. This higher level of nLF at the onset of high‐intensity isometric exercise is in agreement with the greater nLF level previously reported during moderate‐intensity dynamic exercise (Cottin et al., 2004). The difference in the nLF findings providing support for the hypothesis regarding the SNS influence on cardiac rhythm at the onset of exercise isometric while aLF values decreased can be reflected by the lower total power in the 70% compared to the 0% MVC trial due to the performance of exercise. With these nHF and nLF findings supporting these hypotheses, this is especially the case regarding the nLF values that are understood to reflect sympathetic modulation of cardiac rhythm more closely than aLF values due to the overall PNS influence (Malliani et al., 1991; Task Force Report, 1996).

### 4.4 Potential sex differences in HRV power

Based on visual interpretation, responses of all HRV variables during the onset of isometric exercise were similar between male and female subjects, with the possible exception of LF/HF (Figure 8c). In male subjects, LF/HF increased greatly during the first 15 s of the 30‐s exercise and gradually decreased toward the baseline value during the remainder of the 30‐s exercise, while the increase in LF/HF across these time points was much smaller in female subjects. This difference may be due to female individuals exhibiting greater vagal activity overall, which may explain why LF/HF did not increase appreciably in female subjects (Koenig & Thayer, 2016). This result reveals an area of potential future investigation regarding possible sex differences in the autonomic control of cardiac rhythm at the onset of isometric exercise.

### 4.5 Study limitations

A potential limitation in this study was the lack of control for respiratory rates or tidal volume during exercise. While respiratory movement was monitored, subjects were not instructed to breath at a fixed rate during the 0% or 70% MVC trials. Respiratory‐related fluctuations in RRI can be reflected in the HF band, potentially masking some of the parasympathetic activity during exercise (Weippert, Behrens, Rieger, et al., 2015). With an increase in breathing rate during exercise, HF power declines (Brown et al., 1993), suggesting that parasympathetic activity is not entirely reflected by changes in HF power. While fluctuations in respiratory frequency can affect HRV during exercise, these effects were observed with ECG recordings that were at least 3 min in length. With our exercise period lasting only 30 s, the brevity of this exercise may have reduced the influence of respiration on HRV parameters.

## 5 CONCLUSION

In conclusion, the reduction in the PNS influence on cardiac rhythm begins sooner than the augmentation of the SNS influence on cardiac rhythm at the onset of isometric exercise in healthy young adults. These findings further improve our understanding of the autonomic influences on cardiac rhythm at the onset of isometric exercise in healthy young adults, a time when rapid adjustments to HR regulation are occurring. Our results provide clinically relevant information for future areas of investigation in this important area that could include examining the effects of exercise intensity, duration, and/or modality on the autonomic control of cardiac rhythm in healthy populations as well as older populations and patients with cardiovascular disease or autonomic dysfunction. Given the ability of CWT to analyze HRV from non‐stationary signals, especially during short durations of time, future studies could examine the autonomic influences on HRV at times of acute physiological stress that could reveal clinically relevant information regarding cardiovascular disease and autonomic dysfunction when autonomic activity is known to be altered.

## AUTHOR CONTRIBUTIONS

Rachel C. Drew conceived and designed the research. Tyler E. Oliver, Miguel E. Sánchez‐Hechavarría, Ramón Carrazana‐Escalona, and Rachel C. Drew analyzed the data, and all authors interpreted the results of the experiments. Tyler E. Oliver and Rachel C. Drew drafted the manuscript, all authors revised the manuscript, and all authors approved the final version of the manuscript. All authors agree to be accountable for all aspects of the manuscript in ensuring questions related to the accuracy or integrity of any part of the manuscript are appropriately investigated and resolved. All persons designated as authors qualify for authorship and all those who qualify for authorship are listed.

## FUNDING INFORMATION

This study was supported by National Institutes of Health grants P01 HL096570 (LIS) and UL TR000127 (LIS).

## CONFLICT OF INTEREST STATEMENT

The authors declare no competing interests.
